# Circulating Retinol-Binding Protein 4 as a Possible Biomarker of Treatment Response for Ankylosing Spondylitis: An Array-Based Comparative Study

**DOI:** 10.3389/fphar.2020.00231

**Published:** 2020-03-10

**Authors:** Jialing Wu, Xinyu Wu, Zena Chen, Qing Lv, Mingcan Yang, Xuqi Zheng, Qiuxia Li, Yanli Zhang, Qiujing Wei, Shuangyan Cao, Xiaomin Li, Jun Qi, Minjing Zhao, Zetao Liao, Zhiming Lin, Jieruo Gu

**Affiliations:** Department of Rheumatology and Immunology, The Third Affiliated Hospital of Sun Yat-sen University, Guangzhou, China

**Keywords:** ankylosing spondylitis, adalimumab, protein arrays, retinol-binding protein 4, biomarker

## Abstract

**Objective:**

To explore proteins associated with ankylosing spondylitis (AS) and to investigate potential proteins that may predict treatment response of adalimumab (ADA) in AS patients.

**Methods:**

In the discovery cohort, 39 AS patients and 20 healthy controls (HCs) were included, and 16 AS patients received ADA treatment for 24 weeks after included. In the validation cohort, 43 AS patients and 39 HCs were enrolled, and all 43 patients received ADA treatment after enrollment. Blood samples and clinical information were collected from two cohorts at baseline from all participants and week 24 from patients received ADA treatment. A human antibody array containing 1,000 proteins was used in the discovery phase, and Elisa kits were used for protein validation.

**Results:**

Compared with HCs, we identified 53 differentially expressed proteins (DEPs) in AS patients. Bioinformatics analysis revealed they were mostly enriched in coagulation function-related pathways, acute response signaling, and LXR/RXR activation. Bone metabolism pathways were also associated. Comparison between samples of pre- and post-ADA treatment revealed 42 DEPs. They were mostly associated with bone metabolism and inflammation response pathways. Significant enrichment was also found in LXR/RXR activation but not the coagulation function-related pathways. Upstream regulator analysis suggested that most regulators also significantly functioned under usage of ADA. Precisely, seven proteins were abnormally expressed in AS and restored after ADA treatment. Retinol-binding protein 4 (RBP4), one of the seven proteins, was validated that its baseline levels were inversely correlated with improvements in Ankylosing Spondylitis Disease Activity Score-C-reactive protein (ASDAS-CRP). Likewise, percentage changes in RBP4 levels were inversely correlated with changes in ASDAS-CRP score.

**Conclusion:**

A dysregulated serum protein profile existed in AS. ADA exerted a considerable but not entire alteration toward the dysregulation. RBP4 could be a biomarker for predicting and monitoring ADA treatment response.

## Introduction

Ankylosing spondylitis (AS), a subset of axial spondyloarthritis (SpA), is a chronic inflammatory disorder with a predilection for the axial skeleton, characterized by sacroiliitis, uveitis, enthesitis, and spinal inflammation. In severe cases, it can lead to the complete fusion of the axial skeleton (Taurog et al., 2016), causing disability and reduced quality of life. The etiology of AS remains poorly understood, it is thought to be immune-mediated with a complex interplay of both genetic and environmental factors (Ranganathan et al., 2017). Currently, the pathological process of AS is widely considered to include early inflammation, subsequent pathological new bone formation, and eventual ankyloses (Lories and Schett, 2012).

In the past two decades, the introduction of tumor necrosis factor (TNF) α inhibitors has dramatically improved the treatment of AS, especially in patients with insufficient response to conventional therapy. Adalimumab (ADA), a fully humanized monoclonal antibody against TNF-α, can not only reduce symptoms and signs of the disease but also diminish magnetic resonance imaging-detectable inflammation in the sacroiliac joints and spine. Despite the effect on controlling joint inflammation, whether ADA or other TNF inhibitors can inhibit radiographic progression is still controversial (Molnar et al., 2018). Likewise, approximately 40% of patients treated with TNF-α inhibitor therapy fail to achieve favorable clinical improvement (Sieper and Poddubnyy, 2017). Given the uncertainty of therapeutic effects and high costs, it is essential to identify biomarkers for predicting and monitoring the response of ADA treatment.

Proteins are often the effectors of diseases and the targets of treatments. Protein profiling has become a useful technology for clinical biomarker identification, pathogenesis investigation, and new drug discovery (Siu et al., 2009). Several proteomic studies have revealed dysregulation of protein expression by comparing proteomic profiles in AS patients and healthy controls (HCs). The samples were various, including sera, peripheral blood mononuclear cells, and primarily cultured fibroblast cells from ligament biopsies, and mass spectrometry methods or protein arrays were widely applied (Li et al., 2010a, b; Fischer et al., 2012; Wright et al., 2012; Huang et al., 2013; Xu et al., 2015). Although all these studies illustrated dysregulated proteins in AS patients, the disease-associated pathways and proteins were discrepant. This suggests that the proteomic alteration in AS is complicated and remains much to explore. Meanwhile, to our knowledge, the effect of TNF-α inhibitors on serum proteomics in AS has never been studied.

The primary objective of our study was to identify proteins and pathways involved in AS; meanwhile, we anticipated to unveil the effects of ADA treatment on the proteomic profile of AS. Furthermore, to optimize the use of ADA treatment, we aimed to find out proteins that could serve as biomarkers for predicting and monitoring treatment response in AS patients.

## Materials And Methods

### Population and Sample Collection

**FIGURE 1:**
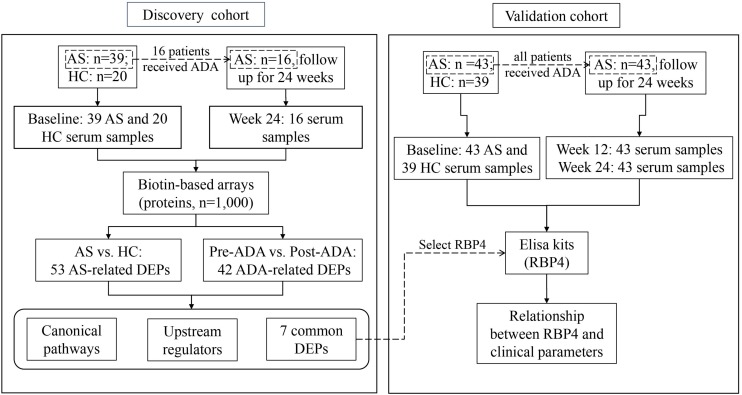
Flow chart of the study. AS, ankylosing spondylitis; HC, healthy control; ADA, adalimumab; DEP, differentially expressed protein; RBP4, retinol-binding protein 4.

The study population was recruited in two phases at the department of Rheumatology and Immunology in the Third Affiliated Hospital of Sun Yat-sen University. In the discovery phase, 39 patients with established AS and 20 age- and sex-matched HCs were included. In this cohort, 16 patients were treated with ADA for 24 weeks according to their disease activity and treatment recommendation after inclusion. In the validation phase, 43 AS patients and 39 age- and sex-matched HCs were included. All 43 patients in the validation cohort accepted ADA therapy for 24 weeks after included (Figure 1). Concomitant medications were allowed as usual during ADA therapy. All eligible patients were over 18 years old and fulfilled the modified New York criteria for AS (van der Linden et al., 1984). All eligible participants were free from infectious diseases, pregnancy, malignancies, and other chronic diseases.

Blood samples were collected from all eligible participants after included. Moreover, blood samples were also obtained from 16 AS patients in the discovery cohort after 24-week treatment with ADA; and were obtained from 43 patients in the validation cohort after 12 and 24 weeks of therapy with ADA. Clinical information was collected at the same day of blood sample collection, including C-reactive protein (CRP), Bath Ankylosing Spondylitis Disease Activity Index (BASDAI), Bath Ankylosing Spondylitis Functional Index (BASFI), Ankylosing Spondylitis Disease Activity Score-CRP (ASDAS-CRP), Maastricht Ankylosing Spondylitis Enthesitis Score (MASES), and Bath Ankylosing Spondylitis Metrology Index (BASMI). Treatment response was evaluated according to the change of ASDAS-CRP (ΔASDAS-CRP). ΔASDAS-CRP ≥ 2 was defined as major improvement of disease (Machado et al., 2011).

Totally 75 serum samples for discovery and 168 serum samples for validation were obtained. All blood samples were collected in the morning after fasting for 8 h. Serum samples were obtained after blood centrifugation at 1,500 r/min for 10 min at −4°C. All the sera were aliquoted and stored at −80°C until the further experiment.

This study was approved by the Ethics Committee of the Third Affiliated Hospital of Sun Yat-sen University. Written informed consent was obtained from all participants for research use and publication of their data before study inclusion.

### Antibody Array Assay

Biotin label-based human antibody array including a total of 1,000 human soluble proteins [RayBio^®^ Label-based (L-Series) Human Antibody Array 1000; RayBiotech, Norcross, GA, United States] was applied for discovery in the 75 serum samples from the discovery cohort. Test operation was conducted following the recommended protocol. Signals were scanned at a wavelength of 532 nm using an InnoScan 300 Microarray Scanner (Innopsys, Carbonne, France; resolution, 10 μm) and were analyzed using RayBio Analysis Tool software (AAH-BLG-1-SW and AAH-BLG-2-SW; RayBiotech, Inc.). Final spot intensities were measured as the original intensities subtracting the background. Data normalization was based on positive and negative controls in each slide.

### Validation of Protein Levels by ELISA

In the validation cohort, retinol-binding protein 4 (RBP4) was detected using ELISA kits (ELH-RBP4-1, RayBiotech, Inc., Norcross, GA, United States) according to the manufacturer’s protocol. First, standard and samples were pipetted into appropriate wells and incubated for 2.5 h at room temperature with gentle shaking. After washing the wells for four times with washing buffer, a prepared biotinylated antibody was added into each well to capture the target protein. After washing away unbound biotinylated antibody, horseradish peroxidase-conjugated streptavidin was pipetted to the wells and bind to the antibody. Subsequently, the wells were washed over. A 3,3,5,5′-tetramethylbenzidine substrate solution was added to the wells, and the color developed in proportion to the amount of RBP4 bound in the sample. Finally, the stop solution was added. The intensity of the color was measured at 450 nm immediately.

### Bioinformatics Analysis

To identify the interaction among proteins, we conducted bioinformatics analysis using Ingenuity Pathway Analysis (IPA) software (Ingenuity Systems^1^). Information on canonical pathways and upstream regulators for the differentially expressed proteins (DEPs) were further analyzed. The canonical pathways were a collection of pathways manually curated by IPA scientists. Fisher’s exact test was used to calculate *P*-values to determine the statistical probability of association between a set of proteins and the specific canonical pathway. Besides, we used the upstream regulator analysis to identify molecules that may regulate proteins expression.

### Statistical Analysis

Normalization was conducted on raw data before statistical analysis. Comparison of both clinical information and protein concentration between AS and HCs groups were calculated using Student’s *t*-test or Mann–Whitney test based on the data characteristics. Similarly, paired *t*-test or Wilcoxon matched-pairs signed-rank test was used for paired samples. Besides, for statistics from microarray, fold change (FC) values of proteins were calculated to indicate relative expression levels. Any FC value ≥ 1.5 or ≤ 1/1.5 in signal intensity for a univariate analysis between groups was considered a measurable difference in expression. Correlations between RBP4 and selected clinical parameters were calculated by methods of Pearson partial correlation adjusting for age, gender, and disease duration. Any *P*-value less than 0.05 was considered statistically significant.

## Results

### Clinical Characteristics of the Study Population

The clinical characteristics of AS patients and HCs are summarized in Table 1. In the discovery cohort, the majority of patients were male (82.05%). Their mean age at sample collection was 30.13 ± 9.51 years, and the age of onset was 20.81 ± 6.47 years. 80% of HCs were male, with an average age of 29.95 ± 9.15 years. There were no significant differences in age and gender between patients and HCs. Sixteen patients suffered from hip joint involvement, while 14 patients presented with peripheral joint involvement. In the validation cohort, clinical characteristics of AS patients and HCs were similar to that of the discovery cohort. For patients who received ADA, clinical indices such as BASDAI, CRP, and ASDAS-CRP significantly decreased after 24-week treatment (Table 2), manifesting good response of ADA treatment at the group level. Moreover, 12 of 16 patients (75%) obtained major improvement (ΔASDAS-CRP ≥ 2) in the discovery cohort, while 19 of 43 patients (43.18%) did in the validation cohort.

**TABLE 1:** Clinical characteristics of the study population.

	**Discovery cohort**		**Validation cohort**	
	**AS patients (*n* = 39)**	**Healthy controls (*n* = 20)**	**AS patients (*n* = 43)**	**Healthy controls (*n* = 39)**
Age, years (mean ± SD)	30.13 ± 9.51	29.95 ± 9.15^n.s.^	30.81 ± 7.99	30.69 ± 6.61^n.s.^
Male, *n* (%)	32 (82.05%)	16 (80%)^n.s.^	34 (79.07%)	31 (79.49%)^n.s.^
HLA-B27+, *n* (%)	38 (97.44%)	–	41 (95.35%)	–
Age of onset, years (mean ± SD)	20.81 ± 6.47	–	18.92 ± 4.32	–
Disease duration, year (mean, IQR)	7 (2, 10)	–	10 (4, 18)	–
Hip joint involvement, *n* (%)	16 (41.03%)	–	21 (48.84%)	–
Peripheral joint involvement, *n* (%)	14 (35.90%)	–	18 (41.86%)	–
Uveitis, *n* (%)	9 (23.08%)	–	9 (20.93%)	–
NSAID use (%)	30 (76.92%)	–	34 (79.07%)	–
DMARD use (%)	14 (35.90%)	–	6 (13.95%)	–
BASDAI (mean ± SD)	5.14 ± 1.54	–	5.23 ± 0.97	–
BASFI (mean ± SD)	4.15 ± 1.93	–	4.22 ± 1.84	–
CRP, mg/L (mean, IQR)	24.80 (9.40, 32.20)	–	17.7 (5.7, 32.8)	–
ESR, mm/h (mean, IQR)	33 (15,47)	–	24 (9, 48)	–

*n.s.: not significant. AS: ankylosing spondylitis; NSAID: non-steroidal anti-inflammatory drug; DMARD: disease-modifying antirheumatic drug; BASDAI: Bath Ankylosing Spondylitis Disease Activity Index; BASFI: Bath Ankylosing Spondylitis Functional Index; CRP: C-reactive protein; ESR: erythrocyte sedimentation rate.*

**TABLE 2:** Clinical features of the AS patients treated with adalimumab.

	Discovery cohort (*n* = 16)		Validation cohort (*n* = 43)		
	Baseline	Week 24	Baseline	Week 12	Week 24
CRP (mg/L) (mean ± SD)	23.79 ± 20.46	3.49 ± 6.53****	23.89 ± 23.90	6.35 ± 11.95****	6.46 ± 12.49****
BASFI (mean ± SD)	4.24 ± 2.18	1.76 ± 1.87**	4.22 ± 1.84	2.72 ± 1.70****	2.34 ± 1.58****^§^
BASDAI (mean ± SD)	5.83 ± 1.14	1.98 ± 1.69****	5.23 ± 0.97	2.77 ± 1.36****	2.41 ± 1.17****^§^
ASDAS-CRP (mean ± SD)	3.71 ± 0.70	1.25 ± 1.01****	3.48 ± 0.76	1.72 ± 0.92****	1.68 ± 0.96****
BASMI (mean ± SD)	2.81 ± 2.24	1.43 ± 1.87**	3.42 ± 2.54	2.84 ± 2.58****	2.74 ± 2.53****
MASES (mean ± SD)	0.38 ± 0.70	0.25 ± 0.56	0.70 ± 1.52	0.17 ± 0.65**	0.26 ± 0.85**

*P-value < 0.05; **P-value < 0.01; ***P-value < 0.001; ****P-value < 0.0001. These represent *P*-values at week 12 or 24 compared to baseline. ^§^Significant changes (*P*-values < 0.05) at week 24 compared to week 12. AS: ankylosing spondylitis; CRP: C-reactive protein; BASFI: Bath Ankylosing Spondylitis Functional Index; BASDAI: Bath Ankylosing Spondylitis Disease Activity Index; ASDAS-CRP: Ankylosing Spondylitis Disease Activity Score-CRP; BASMI: Bath Ankylosing Spondylitis Metrology Index; MASES: Maastricht Ankylosing Spondylitis Enthesitis Score.

### Global Properties of Differentially Expressed Proteins in AS

**FIGURE 2:**
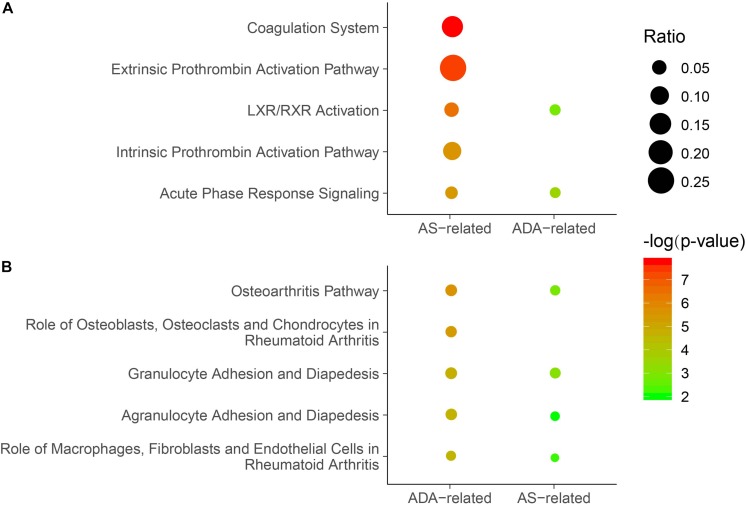
Enriched canonical pathways in which the DEPs were involved. **(A)** The top five canonical pathways identified for the AS-related DEPs. Significance of the pathways based on AS-related or ADA-related DEPs was displayed. **(B)** The top five canonical pathways identified for the ADA-related DEPs. Significance of the pathways based on ADA-related or AS-related DEPs was shown. *P*-values were calculated using Fisher’s exact test. Non-significant *P*-values (*P*-value ≥ 0.05) were not shown. Dots represent the ratio of the number of DEPs to the total number of proteins in each pathway. DEP, differentially expressed protein; AS, ankylosing spondylitis; ADA, adalimumab.

In the discovery phase, 1,000 proteins were detected and analyzed. First, we investigated the differences between AS patients and HCs. In total, 53 DEPs were identified, including 33 proteins with higher expression and 20 proteins with lower expression in AS patients when compared to HCs (Supplementary Table S1). The DEPs and the associated analysis results were defined as AS-related. Based on these DEPs, the pathway analysis was conducted by IPA software. The top five canonical pathways comprised coagulation-related pathways (Coagulation system, extrinsic prothrombin activation pathway and intrinsic prothrombin activation pathway), LXR/RXR activation, and acute phase response signaling (Figure 2A and Supplementary Table S2). Additionally, significant enrichment was also found in bone metabolism-related pathways, including osteoarthritis pathway (Figure 2B and Supplementary Table S2). Moreover, we used upstream regulator analysis to predict the potential molecules responsible for differential protein expression in AS. The top 10 most significantly identified upstream regulators predominantly play roles in inflammation and immune system, such as transforming growth factor-beta 1 (TGF-B1), TNF, lipopolysaccharide, and interleukin-1 (IL-1) (Table 3 and Supplementary Table S3). Notably, two regulators, Forskolin and cyclic adenosine monophosphate (cyclic AMP), are associated with bone resorption (Lerner et al., 1986).

**TABLE 3:** Top predicted upstream regulators of AS-related or ADA-related DEPs.

Upstream regulator	*P*-value	
	AS-related	ADA-related
Diethylstilbestrol^a^	2.21E-09	0.0203
Forskolin^a^	6.73E-09	0.0443
Cyclic AMP^a^	9.11E-09	0.000146
Lipopolysaccharide ^a^	1.3E-08	4.53E-06
Phorbol myristate acetate^a^	4.63E-08	0.000052
**Dexamethasone**^a,b^	**8.08E-08**	**8.29E-09**
**Beta-estradiol**^a,b^	**1.15E-11**	**1.81E-07**
**TGFB1**^a,b^	**1.36E-09**	**4.22E-07**
**IL1**^a,b^	**9.46E-08**	**2.53E-07**
**TNF**^a,b^	**1.09E-08**	**1.22E-08**
IL1B^b^	1.09E-07	8.85E-09
SP1^b^	6.65E-07	6.04E-08
Cholecalciferol^b^	0.000169	8.28E-08
IL6^b^	1.44E-06	1.41E-07
TWIST1^b^	0.00596	4.07E-07

*^*a*^AS-related top 10 upstream regulators; ^*b*^ADA-related top 10 upstream regulators. Common top upstream regulators were marked in bold. AS: ankylosing spondylitis; ADA, adalimumab.*

### Systemic Alteration Caused by ADA Treatment

Subsequently, we analyzed the differential expression of proteins in samples from AS patients pre- and post-treated with ADA for 24 weeks. We identified a total of 42 DEPs, of which 32 and 10 proteins were up- and down-regulated, respectively (Supplementary Table S4). These DEPs and the associated analysis results were defined as ADA-related. The top five canonical pathways analyzed by IPA software mainly act on bone metabolism (Osteoarthritis pathway and role of osteoblasts, osteoclasts and chondrocytes in rheumatoid arthritis) and inflammatory response (granulocyte adhesion and diapedesis, agranulocyte adhesion and diapedesis, and role of macrophages, fibroblasts, and endothelial cells in rheumatoid arthritis) (Figure 2B and Supplementary Table S2). All these pathways, except role of osteoblasts, osteoclasts, and chondrocytes in rheumatoid arthritis, had significant *P*-values in the analysis of AS patients versus HCs. In other words, most of the top significant ADA-related pathways were AS-related. However, among the top five AS-related canonical pathways, three coagulation function-related pathways were not significantly ADA-related (Figure 2A). The upstream regulator analysis revealed that five of the top 10 ADA-related regulators also ranked top 10 in AS, including TNF, TGF-B1, and IL-1 (Table 3 and Supplementary Table S3). For the rest five AS-related regulators, they were all ADA-related, and vice versa. Given this, ADA treatment may target and alter the dysregulation in AS through these molecules.

### Identification of Pivotal Proteins Associated With AS and ADA Therapy

**FIGURE 3:**
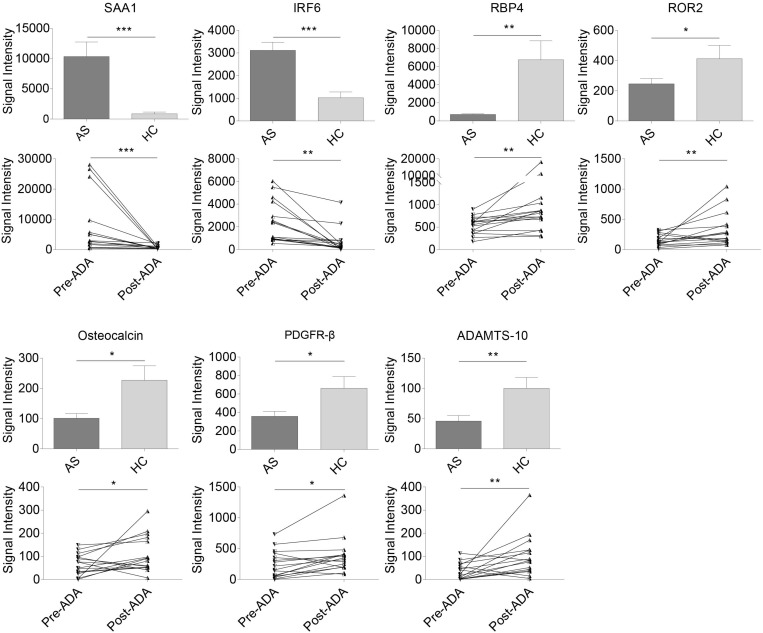
Relative expression levels in seven pivotal DEPs among samples from all AS patients, HCs, patients pre- and post-treated with ADA in the discovery cohort. Two proteins (SAA1 and IRF6) were expressed higher in AS group and down-regulated after 24-week ADA treatment, and five proteins (RBP4, ROR2, osteocalcin, PDGFR-β, and ADAMTS-10) were expressed lower and up-regulated after ADA treatment. Data were shown as box and whisker plots (mean ± SEM) and before-after plots for pair samples. *P-value < 0.05, **P-value < 0.01, ***P-value < 0.001. DEP, differentially expressed protein; HC, healthy control; AS, ankylosing spondylitis; ADA, adalimumab; Pre-ADA, pre-treated with ADA; Post-ADA, post-treated with ADA.

Of the 53 AS-related DEPs, seven proteins were also identified as ADA-related in the discovery phase (Figure 3). These seven proteins included Serum amyloid A-1 (SAA1), Interferon regulatory factor 6 (IRF6), RBP4, Tyrosine-protein kinase transmembrane receptor (ROR2), Osteocalcin, Platelet-derived growth factor receptor beta (PDGFR-β), and A disintegrin and metalloproteinase with thrombospondin motifs 10 (ADAMTS-10). SAA1 and IRF6 were expressed higher in the AS group compared with HCs and had decreased after 24-week ADA treatment. On the contrary, levels of RBP4, ROR2, Osteocalcin, PDGFR-β, and ADAMTS-10 were lower in AS patients than that of HCs and had increased after ADA therapy. Significant differences in these protein levels at baseline between HCs and the 16 patients treated with ADA were also found (Supplementary Figure S1). We also conducted subgroup analyses to evaluate whether these proteins were implicated with peripheral joint involvement and hip joint involvement of AS (Supplementary Tables S5, S6). Precisely, SAA1 and IRF6 levels were higher in patients with peripheral joint involvement than in patients without peripheral joint involvement. No significant differences were found in subgroup AS patients in terms of hip joint involvement. Remarkably, only results of SAA1, IRF6, and RBP4 from subgroup AS patients-versus-HCs analyses were consistent with that from overall AS patients-versus-HCs analyses. To note, RBP4 was the second most DEP based on FC values among the seven proteins, second only to SAA1. Furthermore, RBP4 is an adipokine associated with inflammation, bone metabolism, as well as lipid metabolism (Koch et al., 2010; Hatfield et al., 2012), which are crucial in the pathogenesis of AS. Taken together, we thought of RBP4 as the most promising protein and chose it for further analysis.

### Identification of RBP4 as a Biomarker for Predicting and Monitoring Treatment Response

**FIGURE 4:**
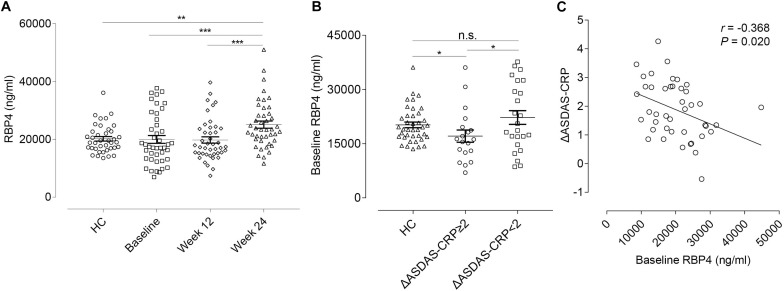
RBP4 expression levels and the correlation with ΔASDAS-CRP in the validation cohort. **(A)** Relative serum RBP4 expression levels between HCs and AS patients at baseline, week 12, and week 24. Data were shown as scatter plots (mean ± SEM). **(B)** Relative baseline RBP4 expression levels in HCs and in AS patients with and without major improvement in ASDAS-CRP (ΔASDAS-CRP ≥ 2 and ΔASDAS-CRP < 2) at week 24. Data were shown as scatter plots (mean ± SEM). **(C)** Linear correlation between baseline RBP4 levels and ΔASDAS-CRP at week 24. *P-value < 0.05, **P-value < 0.01, ***P-value < 0.001. ΔASDAS-CRP, improvements of Ankylosing Spondylitis Disease Activity Score-C-Reactive Protein; HC, healthy control; AS, ankylosing spondylitis; n.s., not significant.

We further tested serum levels of RBP4 in the validation cohort. At this stage, the RBP4 level was found to be up-regulated after ADA treatment, which was consistent with the result found in the discovery cohort. Nevertheless, there was no significant difference between overall AS patients and HCs in RBP4 levels at baseline (Figure 4A). In this regard, we conducted further subgroup analyses. No significant differences were found for RBP4 levels in subgroup AS patients regarding hip and peripheral joint involvement. Interestingly, patients who achieved major improvements (ΔASDAS-CRP ≥ 2, *n* = 19) after 24-week ADA treatment had lower baseline levels in RBP4, compared with HCs as well as patients who did not reach major improvements (*n* = 24) (*P* = 0.015 and *P* = 0.049, respectively) (Figure 4B). After adjustment for gender, age, and disease duration, an inverse correlation (*r* = -0.368, *P* = 0.020) existed between baseline serum RBP4 levels and ΔASDAS-CRP at week 24 (Figure 4C), indicating the lower baseline RBP4 level, the greater the improvement in ASDAS-CRP. Therefore, baseline RBP4 levels could serve as a predictor for ADA treatment response.

Furthermore, we conducted correlation analyses to evaluate whether changes in RBP4 paralleled that in ASDAS-CRP (Table 4). After adjustment for gender, age, and disease duration, from baseline to week 12, percentage changes in RBP4 levels were inversely correlated with changes in ASDAS-CRP scores (*r* = -0.547, *P* < 0.001). Similarly, percentage changes in RBP4 levels had an inverse correlation with changes in ASDAS-CRP from baseline to week 24 (*r* = -0.491, *P* = 0.001). In other words, the serum level in RBP4 was shown to change along with ASDAS-CRP score. No similar associations were found with BASFI, BASDAI, BASMI, and MASES scores.

**TABLE 4:** Correlations between percentage changes in RBP4 levels and changes in clinical parameters from baseline to week 12 and week 24.

	Baseline to week 12		Baseline to week 24	
	*r*	*P*	*r*	*P*
ΔCRP^†^	−0.335	0.035*	−0.451	0.003**
ΔASDAS-CRP	−0.547	0.000***	−0.491	0.001**
ΔBASFI	−0.308	0.053	−0.118	0.468
ΔBASMI	−0.252	0.116	−0.400	0.011*
ΔBASDAI	−0.314	0.048*	−0.217	0.178
ΔMASES	−0.440	0.005**	−0.178	0.271

^†^Changes in CRP levels were calculated as percentage changes. *P-value < 0.05; **P-value < 0.01; ***P-value < 0.001. CRP: C-reactive protein; ASDAS-CRP: Ankylosing Spondylitis Disease Activity Score-CRP; BASFI: Bath Ankylosing Spondylitis Functional Index; BASMI: Bath Ankylosing Spondylitis Metrology Index; BASDAI: Bath Ankylosing Spondylitis Disease Activity Index; MASES: Maastricht Ankylosing Spondylitis Enthesitis Score.

## Discussion

In this study, first, we described a systemic profile of dysregulated serum proteins in AS. Subsequently, we illustrated the effects of ADA treatment on the protein profile. Seven proteins were expressed at abnormal levels in AS patients and restored after ADA treatment. Additionally, our study revealed that RBP4 could serve as a biomarker for predicting and monitoring response to ADA treatment in AS.

In the present study, we demonstrated that serum expressions of 53 proteins in AS differed from those in HCs based on a human antibody array containing 1,000 proteins. A previous study by Fischer et al. (2012) identified a total of 316 proteins in serum using nano-liquid chromatography/mass spectrometry analysis of serum samples from AS and HCs. They identified 22 proteins playing roles in innate immunity and the acute phase inflammatory response were up- or down-regulated in AS. When comparing the results of this research with ours, Transferrin was the only DEP replicated in our study. The discrepancy may result from several divergences between the two studies. First, owing to the different methods of protein detection, some DEPs discovered in Fischer’s research were not detected in our study. Second, the selection criteria were different; for example, consistent with Fischer’s research, *P*-value of Thrombospondin-1 was less than 0.05 in our study, but we excluded it for its FC value. Third, the study population were different; all recruited members were European ethnicities in Fischer’s research and Chinese in our study.

Furthermore, we identified 42 proteins that were significantly changed after ADA treatment. Globally, ADA exerted considerable but not entire influence on AS. The bioinformatics analysis showed that acute phase response signaling pathway was involved in AS and could be modified by ADA treatment, which reinforced the hypothesis that acute inflammation plays a vital role in the pathogenesis of AS and ADA can retard the progress of the disease. LXR/RXR activation was also a top-ranked pathway in AS and was altered by ADA, which plays an imperative role in lipid metabolism and inflammation (Tontonoz and Mangelsdorf, 2003). Moreover, it is not neglectable that the coagulation pathways, which are associated with autoimmune and inflammatory disorders (Brouwer et al., 2004; Gobel et al., 2018), were also AS-related. Recently, a large population-based cohort study disclosed AS was associated with a higher risk of venous thromboembolism (VTE) compared with people without AS (Avina-Zubieta et al., 2019). Herein, our results suggest that an abnormal coagulation status exists in AS and may contribute to the increased VTE risk. However, ADA was not shown to have effects on coagulation processes in our data. That ADA increases or decreases the VTE risk needs further study. Besides, the analysis of potential upstream regulators suggested that all top 10 molecules probably induced dysregulation in AS patients were shown to function under ADA treatment. Five molecules had high significances in both analyses. These molecules comprised TNF, IL-1, and TGF-B1, which were well-known for their associations with AS (Archer, 1995; Orman et al., 2002; Haibel et al., 2005).

In particular, compared with HCs, seven proteins were identified to be differentially expressed in AS and modified by ADA therapy. In previous studies, SAA1 was confirmed to correlate with disease activity in AS and demonstrated to probably assist in the monitoring of the efficacy of anti-TNF treatment in AS (Lange et al., 2000; Jung et al., 2007; de Vries et al., 2009). IRF6 was reported to selectively promote expression of TLR3-inducible IL-23p19 and inhibit expression of TLR3-inducible IFN-β in human keratinocytes (Ramnath et al., 2015), suggesting its effects on IL-23 production. ROR2 shows more possibility about bone metabolism, in that Wnt5a-ROR2 pathway is crucial for osteogenic differentiation, which may be relevant in the pathological process of new bone formation in SpA (Gonzalez-Chavez et al., 2016). Previous studies on osteocalcin levels in AS demonstrated contradictory results (Visvanathan et al., 2009; Pedersen et al., 2011a, b; Kwon et al., 2012), while levels of osteocalcin were significantly lower in AS patients in our study. Besides, osteocalcin was shown to increase after anti-TNF-α therapy (Pedersen et al., 2011a), which is consistent with our results. Osteoblasts stimulate osteoclastogenesis in a PDGFR-β-dependent manner (Bartelt et al., 2018), suggesting that PDGFR-β is related to bone development. Similar to matrix metalloproteinase-3 (MMP3), a useful serum marker of disease activity in AS patients (Danve and O’Dell, 2015), ADAMTS-10 is one of the primary enzymes that cleave collagen and proteoglycans. Both of them are involved in the process of disc degeneration, with MMP-3 up-regulated and ADAMTS-10 down-regulated in degenerative intervertebral disc tissue (Wang et al., 2015). In a whole, most of these proteins are involved in either inflammation or bone metabolism process.

RBP4 belongs to the lipocalin family and is the specific carrier for retinol in the circulation. RBP level was reported to be lower in sera of 40 AS patients than in 46 controls, with almost 60% of the patients had active disease (O’Shea et al., 2007). In another study, Genre et al. (2014) described a non-significant difference in RBP4 serum levels compared with controls and a significant decrease 2 h after infliximab infusion. In our data, RBP4 was significantly lower in AS patients compared with HCs in the discovery cohort. In the validation cohort, there was no significant difference between overall patients and HCs. Instead, there existed significantly lower levels in RBP4 in a subgroup of AS patients who had a favorable response to ADA treatment. It could be explained to some extent in that most patients (75%) that received ADA in the discovery cohort had major improvement while no more than half of the patients (43.18%) did in the validation cohort. In this context, it is consistent with our results of RBP4 being a predictor of improvements in ADSAS-CRP. Thus, differences between results from previous and this study may be due to individual variance, especially their response to treatment. For Genre’s research, RBP4 was detected soon after infliximab injection rather than a long period of therapy, implying this reduction might be temporary. Both discovery and validation analysis showed a significant upregulation of RBP4 after treatment in the present study. Besides, RBP4 could have the potential for discriminating against inflammatory disorders. The previous study showed that RBP4 levels were higher in moderate-severe psoriasis patients, and 6-month ADA treatment significantly reduced RBP4 levels (Romani et al., 2013; Pina et al., 2015), right opposing the results in AS according to our data.

In the current study, as mentioned before, baseline levels in RBP4 can serve as a predictor for improvements in ASDAS-CRP from baseline to week 24. Furthermore, from baseline to week 12 or week 24, percentage changes in RBP4 levels were inversely correlated with changes in ASDAS-CRP score, indicating serum RBP4 levels could play a role in monitoring disease during the treatment. Unlike CRP levels which significantly increased in the first 12 weeks (Table 2), significant changes in RBP4 levels occur from week 12 to week 24. This finding suggests that RBP4 may potentially play a downstream role in the inflammatory cascade. In this regard, it is noteworthy that RBP4 is thought to be involved in bone metabolism (Hogstrom et al., 2008; Hatfield et al., 2012). Whether RBP4 works as a mediator connecting inflammation and subsequent abnormal bone formation in AS is worth further exploration. Taken together, RBP4 has the potential to be a biomarker for treatment response.

Nevertheless, and notably, what we found is conclusively in serum and suggests a systemic change in peripheral blood. Whether the same events exist in local sites needs more investigations.

## Conclusion

In conclusion, we illustrated an overview of dysregulation of serum proteins in AS patients and the changes caused by ADA treatment. In our data, most AS-related pathways and upstream regulators were associated with ADA treatment. Coagulation pathways seemed to be crucial in AS but not to be altered by ADA. Moreover, we identified seven potential disease-associated proteins for diagnostics or therapeutics. In particular, RBP4 was demonstrated to be a candidate biomarker for predicting and monitoring the response to ADA treatment.

## Data Availability Statement

The datasets used and/or analyzed in the current study are available from the corresponding author upon reasonable request.

## Ethics Statement

The studies involving human participants were reviewed and approved by the Ethics Committee of the Third Affiliated Hospital of Sun Yat-sen University. The patients/participants provided their written informed consent to participate in this study.

## Author Contributions

JG designed the study and provided funding. JQ, ZEL, ZHL, and QL designed the study conception and collected patients. MZ, SC, MY, XL, and YZ collected the blood samples and obtained the sera. XZ, QW, and QIL collected and rearranged patients’ information. ZC and XW provided support for the bioinformatics analyses. JW conducted ELISA, interpreted and analyzed the data, and wrote the first draft. JG corrected the manuscript. All authors approved the final manuscript.

## Conflict of Interest

The authors declare that the research was conducted in the absence of any commercial or financial relationships that could be construed as a potential conflict of interest.

## Footnotes

1 http://www.INGENUITY.com
