# Supplementary material for: Circulating Retinol-Binding Protein 4 as a Possible Biomarker of Treatment Response for Ankylosing Spondylitis: An Array-Based Comparative Study
Source: Front Pharmacol. 2020 Mar 10;11:231. doi: 10.3389/fphar.2020.00231 (PMC7076136; doi:10.3389/fphar.2020.00231)
Supplement: Supplementary file 1 [file Image_1.pdf]

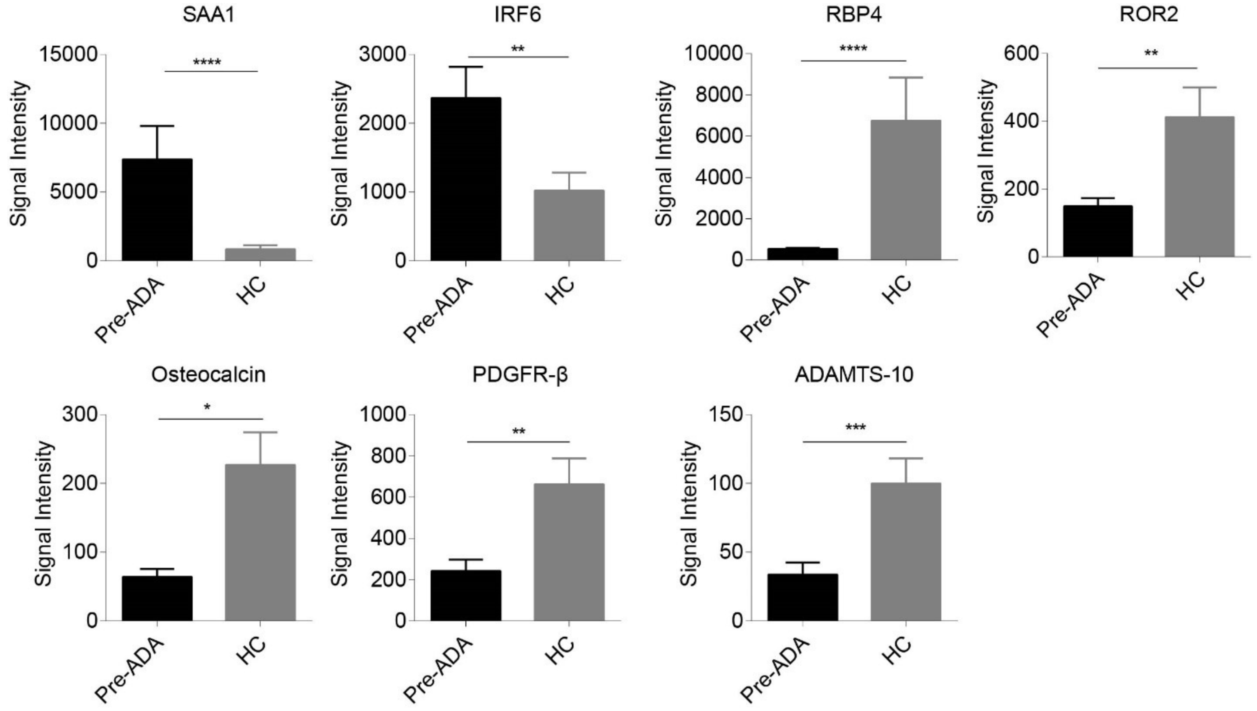

Figure S1 Relative expression levels in seven pivotal DEPs between HCs and 16 AS patients pre-treated with ADA in the discovery cohort. Two proteins (SAA1 and IRF6) were expressed higher, and five proteins (RBP4, ROR2, osteocalcin, PDGFR-β, and ADAMTS-10) were expressed lower in the AS group. Data were presented as box and whisker plots (mean ± SEM). **P* value < 0.05, ** *P* value < 0.01, *** *P* value < 0.001, **** *P* value < 0.0001. DEP, differentially expressed protein; HC, healthy control; AS, ankylosing spondylitis; ADA, adalimumab; Pre-ADA, pre-treated with ADA.
