# Supplementary material for: Circulating Retinol-Binding Protein 4 as a Possible Biomarker of Treatment Response for Ankylosing Spondylitis: An Array-Based Comparative Study
Source: Front Pharmacol. 2020 Mar 10;11:231. doi: 10.3389/fphar.2020.00231 (PMC7076136; doi:10.3389/fphar.2020.00231)
Supplement: Supplementary file 2 [file Table_1.PDF]

Table S1. Differentially expressed proteins identified between AS patients and healthy controls

Protein name (a.k.a.*)	P value	FC	log2FC	up/down-regulation
SAA1	<0.001	12.159	3.604	↑
Claudin-4 (CLDN4)	0.003	3.273	1.711	↑
IRF6	<0.001	3.055	1.611	↑
Thrombomodulin (THBD)	0.043	2.441	1.288	↑
NR3C3 (PGR)	<0.001	2.434	1.283	↑
TLR4	0.010	2.339	1.226	↑
SRMS	<0.001	2.328	1.219	↑
Factor XIII A (F13A1)	0.020	2.323	1.216	↑
Inhibin B (INHBB)	0.033	2.231	1.158	↑
CA 19-9	<0.001	2.223	1.153	↑
Plasminogen (PLG)	<0.001	2.005	1.004	↑
RECK	0.005	1.965	0.975	↑
Procalcitonin (CALCA)	<0.001	1.954	0.966	↑
ROBO4	0.016	1.943	0.959	↑
PEPSINOGEN II	0.044	1.887	0.916	↑
Thrombopoietin (TPO)	0.029	1.872	0.904	↑
Glypican 5	0.004	1.869	0.903	↑
HSP20	0.033	1.847	0.885	↑
CD55	0.024	1.836	0.877	↑
ADAMTS-15	0.002	1.829	0.871	↑
TACI (TNFRSF13B)	0.019	1.824	0.867	↑
PSA-Free (KLK3)	<0.001	1.734	0.794	↑
FSH	0.003	1.733	0.794	↑
MMP-20	0.019	1.675	0.744	↑
FGFR1 alpha	0.000	1.626	0.702	↑
BMPR-IB (ALK-6)	0.016	1.598	0.676	↑
TNK1	0.031	1.593	0.672	↑
CCR8	0.009	1.591	0.670	↑
D-Dimer	<0.001	1.580	0.660	↑
Follistatin-like 1 (FSTL1)	0.031	1.578	0.658	↑
Fibrinopeptide A (FGA)	0.001	1.531	0.615	↑
TSH	0.008	1.510	0.594	↑
IL-17F	0.045	1.509	0.594	↑
CD200	0.038	0.654	-0.613	↓
Nestin	0.022	0.618	-0.695	↓
ROR2	0.049	0.581	-0.783	↓
RANTES (CCL5)	0.049	0.558	-0.841	↓
PDGFR-β	0.034	0.541	-0.887	↓
CD90 (THY1)	0.047	0.539	-0.892	↓
Transferrin (TF)	0.004	0.469	-1.093	↓
ADAMTS-10	0.004	0.459	-1.125	↓

Serotonin (SLC6A4)	0.024	0.446	-1.165	↓
Osteocalcin (BGLAP)	0.019	0.445	-1.167	↓
TFPI	0.025	0.441	-1.181	↓
Gas1	0.017	0.321	-1.639	↓
Activin A (INHBA)	0.049	0.302	-1.727	↓
GATA-4	0.006	0.293	-1.770	↓
Livin (BIRC7)	0.013	0.252	-1.986	↓
Serpin A8 (AGT)	<0.001	0.186	-2.427	↓
ESAM	0.002	0.156	-2.685	↓
Pancreatic Polypeptide	0.026	0.109	-3.195	↓
RBP4	0.009	0.107	-3.225	↓
KCC3 (SLC12A6)	0.021	0.020	-5.658	↓

*a.k.a.: also known as.

AS: ankylosing spondylitis; FC: fold change.
