# Supplementary material for: Circulating Retinol-Binding Protein 4 as a Possible Biomarker of Treatment Response for Ankylosing Spondylitis: An Array-Based Comparative Study
Source: Front Pharmacol. 2020 Mar 10;11:231. doi: 10.3389/fphar.2020.00231 (PMC7076136; doi:10.3389/fphar.2020.00231)
Supplement: Supplementary file 3 [file Table_2.PDF]

Table S2. AS-related or ADA-related canonical pathways

Group	Ingenuity Canonical Pathways	-log(p-value)	Ratio	Molecules
AS-related	Acute Phase Response Signaling	5.46	0.0335	AGT,SAA1,TF,FGA,RBP4,PLG
AS-related	Intrinsic Prothrombin Activation Pathway	5.58	0.0952	THBD,KLK3,FGA,F13A1
AS-related	LXR/RXR Activation	6.46	0.0496	TLR4,AGT,SAA1,TF,FGA,RBP4
AS-related	Extrinsic Prothrombin Activation Pathway	7.35	0.25	TFPI,THBD,FGA,F13A1
AS-related	Coagulation System	7.78	0.143	TFPI,THBD,FGA,F13A1,PLG
AS-related	Role of Macrophages, Fibroblasts and Endothelial Cells in Rheumatoid Arthritis	2.17	0.0122	TLR4,FGFR1,CCL5,ROR2
AS-related	Agranulocyte Adhesion and Diapedesis	2	0.0156	CCL5,CLDN4,MMP20
AS-related	Granulocyte Adhesion and Diapedesis	3.11	0.0223	CCL5,CLDN4,MMP20,THY1
AS-related	Osteoarthritis Pathway	2.84	0.0189	BGLAP,TLR4,FGFR1,RBP4
ADA-related	Role of Macrophages, Fibroblasts and Endothelial Cells in Rheumatoid Arthritis	4.61	0.018	IL1RN,RYK,CSF1,SFRP1,ROR2,MMP1
ADA-related	Agranulocyte Adhesion and Diapedesis	4.65	0.0259	CCL22,IL1RN,CCL15,MMP1,MMP14
ADA-related	Granulocyte Adhesion and Diapedesis	4.78	0.0276	CCL22,IL1RN,CCL15,MMP1,MMP14
ADA-related	Role of Osteoblasts, Osteoclasts and Chondrocytes in Rheumatoid Arthritis	5.39	0.0248	BGLAP,IL1RN,CSF1,SFRP1,MMP1,MMP14
ADA-related	Osteoarthritis Pathway	5.64	0.0274	BGLAP,TGFBR2,GDF2,TIMP3,RBP4,MMP1
ADA-related	Acute Phase Response Signaling	3.54	0.0221	MBL2,SAA1,IL1RN,RBP4
ADA-related	LXR/RXR Activation	2.82	0.0234	SAA1,IL1RN,RBP4

AS: ankylosing spondylitis; ADA: adalimumab.
