# Supplementary material for: Circulating Retinol-Binding Protein 4 as a Possible Biomarker of Treatment Response for Ankylosing Spondylitis: An Array-Based Comparative Study
Source: Front Pharmacol. 2020 Mar 10;11:231. doi: 10.3389/fphar.2020.00231 (PMC7076136; doi:10.3389/fphar.2020.00231)
Supplement: Supplementary file 4 [file Table_3.PDF]

Table S3. AS-related or ADA-related upstream regulators

Group	Upstream Regulator	Molecule Type	p-value of overlap	Target molecules in dataset
AS-related	beta-estradiol	chemical - endogenous mammalian	1.15E-11	AGT,BGLAP,BMPR1B,CALCA,CCL5,CD55,CLDN4,FSHB,FSTL1,GATA4,INHBA,INHBB,IRF6,KLK3,PDGFRB,PGR,SAA1,SLC6A4,TF,TFPI,THBD,TLR4,TSHB
AS-related	TGFB1	growth factor	1.36E-09	BGLAP,CALCA,CCL5,CD55,CLDN4,ESAM,F13A1,FGA,FGFR1,GAS1,IL17F,INHBA,INHBB,KLK3,PDGFRB,TFPI,THBD,THPO,THY1,TLR4
AS-related	diethylstilbestrol	chemical drug	2.21E-09	BGLAP,CD200,CD55,GATA4,INHBA,INHBB,KLK3,PDGFRB,PGR,TF
AS-related	forskolin	chemical toxicant	6.73E-09	BGLAP,BIRC7,CALCA,CCL5,CCR8,CD200,CD55,FSHB,GATA4,INHBA,INHBB,KLK3,PGR
AS-related	cyclic AMP	chemical - endogenous mammalian	9.11E-09	BGLAP,CALCA,GATA4,INHBA,PGR,RBP4,TF,THBD,TSHB
AS-related	TNF	cytokine	1.09E-08	AGT,BGLAP,BIRC7,CALCA,CCL5,CCR8,CD200,CD55,CLDN4,FGFR1,INHBA,KLK3,SAA1,SLC12A6,TF,TFPI,THBD,THY1,TLR4
AS-related	lipopolysaccharide	chemical drug	1.3E-08	AGT,CALCA,CCL5,CCR8,CD200,CD55,GAS1,INHBA,INHBB,PGR,PLG,SAA1,TF,TFPI,THBD,THPO,THY1,TLR4,TNFRSF13B
AS-related	phorbol myristate acetate	chemical drug	4.63E-08	AGT,BGLAP,CCL5,CD55,FSHB,IL17F,INHBA,INHBB,KLK3,PDGFRB,PGR,THY1,TLR4,TNFRSF13B,TSHB
AS-related	dexamethasone	chemical drug	8.08E-08	ADAMTS15,AGT,BGLAP,CALCA,CCL5,CD55,FGA,FSTL1,GAS1,INHBA,INHBB,PGC,PGR,RBP4,SAA1,SLC6A4,TF,THBD,TLR4
AS-related	IL1	group	9.46E-08	CCL5,CD55,INHBA,PDGFRB,RBP4,SAA1,THBD,THY1,TLR4

AS-related	IL1B	cytokine	1.09E-07	BGLAP,CALCA,CCL5,CCR8,CD55,F13A1,IL17F,INHBA,SAA1,SLC6A4,THBD,THY1,TLR4
AS-related	SP1	transcription regulator	6.65E-07	BGLAP,CCL5,CD55,FGFR1,NES,PDGFRB,PGR,RECK,ROBO4,THBD
AS-related	cholecalciferol	chemical - endogenous mammalian	0.000169	BGLAP,CCL5,F13A1,PDGFRB
AS-related	IL6	cytokine	1.44E-06	AGT,CALCA,CCL5,FGA,IL17F,KLK3,PLG,SAA1,TF,THPO,TLR4
AS-related	TWIST1	transcription regulator	0.00596	BGLAP,CLDN4,TF
ADA-related	dexamethasone	chemical drug	8.29E-09	ALCAM,BDNF,BGLAP,CSF1,DMP1,IL1RN,MMP1,MMP14,RBP4,SAA1,SCARB2,SFRP1,SIGLEC9,SLC2A2,SLC2A3,TAF4,TGFBR2,TIMP3
ADA-related	IL1B	cytokine	8.85E-09	ACPP,BGLAP,CCL22,CSF1,IL1RN,MMP1,MMP14,NAD+,SAA1,SLC2A2,TGFBR2,TIMP3,TNFSF15
ADA-related	TNF	cytokine	1.22E-08	ALCAM,BDNF,BGLAP,CCL22,CD38,CSF1,IL1RN,MBL2,MMP1,MMP14,PTPRC,SAA1,SFRP1,SLC2A2,TGFBR2,TIMP3,TNFSF15
ADA-related	SP1	transcription regulator	6.04E-08	BDNF,BGLAP,CCL22,CKB,MMP1,MMP14,PDGFRB,SLC2A3,TGFBR2,TIMP3
ADA-related	cholecalciferol	chemical - endogenous mammalian	8.28E-08	BDNF,BGLAP,CD38,CSF1,PDGFRB,TGFBR2

ADA-related	IL6	cytokine	1.41E-07	ADAMTS13,ADGRB1,BDNF,CD38,CSF1,IL1RN,LEFTY2,MMP1,PTPRC,SAA1,SLC2A2
ADA-related	beta-estradiol	chemical - endogenous mammalian	1.81E-07	ACPP,ALCAM,BDNF,BGLAP,CKB,INSL3,IRF6,LEFTY2,MMP1,PDGFRB,PTK2,SAA1,SFRP1,SLC2A3,TGFBR2,TIMP3
ADA-related	IL1	group	2.53E-07	BDNF,CSF1,IL1RN,MMP1,PDGFRB,RBP4,SAA1,SLC2A3
ADA-related	TWIST1	transcription regulator	4.07E-07	ALCAM,BGLAP,LEFTY2,MMP1,TGFBR2,TIMP3
ADA-related	TGFB1	growth factor	4.22E-07	ACPP,ADGRB1,BDNF,BGLAP,CSF1,IL1RN,MMP1,MMP14,PDGFRB,PTK2,PTPRC,SFRP1,SLC2A3,TGFBR2,TIMP3
ADA-related	diethylstilbestrol	chemical drug	0.0203	BGLAP,INSL3,PDGFRB
ADA-related	forskolin	chemical toxicant	0.0443	ACPP,BDNF,BGLAP,CSF1
ADA-related	cyclic AMP	chemical - endogenous mammalian	0.000146	BDNF,BGLAP,IL1RN,MMP1,RBP4
ADA-related	lipopolysaccharide	chemical drug	4.53E-06	ALCAM,BDNF,CCL22,CD38,CRLF2,CSF1,IL1RN,MBL2,MMP1,MMP14,SAA1,SLC2A2,TIMP3,TNFSF15
ADA-related	phorbol myristate acetate	chemical drug	0.000052	BDNF,BGLAP,CSF1,IL1RN,MMP1,MMP14,PDGFRB,PTK2,PTPRC,SLC2A2

AS: ankylosing spondylitis; ADA: adalimumab.
