# Supplementary material for: Circulating Retinol-Binding Protein 4 as a Possible Biomarker of Treatment Response for Ankylosing Spondylitis: An Array-Based Comparative Study
Source: Front Pharmacol. 2020 Mar 10;11:231. doi: 10.3389/fphar.2020.00231 (PMC7076136; doi:10.3389/fphar.2020.00231)
Supplement: Supplementary file 5 [file Table_4.PDF]

Table S4. Differentially expressed proteins identified in AS patients before and after ADA treatment

Protein name (a.k.a.*)	P value	FC	log2FC	up/down-regulation
MIP-1d(CCL15)	0.022	12.501	3.644	↑
RBP4	0.002	4.527	2.179	↑
TGF-beta RII(TGFBR2)	0.007	3.190	1.673	↑
MMP-14	0.041	3.102	1.633	↑
ADAMTS-10	0.002	3.003	1.586	↑
TIMP-3	0.028	2.605	1.381	↑
TAF4	0.005	2.500	1.322	↑
BMP-9(GDF2)	0.030	2.467	1.303	↑
M-CSF(CSF1)	0.020	2.444	1.289	↑
MMP-1	0.035	2.357	1.237	↑
CK-MB(CKB)	0.013	2.239	1.163	↑
TL1A(TNFSF15)	0.013	2.188	1.130	↑
DMP-1	0.036	2.182	1.125	↑
ROR2	0.008	2.155	1.108	↑
ADAMTS-13	0.009	2.154	1.107	↑
ALCAM	0.028	2.145	1.101	↑
IL-1ra (IL1RN)	0.042	2.007	1.005	↑
CD38	0.017	1.873	0.905	↑
Glut3(SLC2A3)	0.028	1.868	0.901	↑
Glut2(SLC2A2)	0.030	1.801	0.849	↑
ACPP	0.012	1.786	0.837	↑
SFRP1	0.013	1.735	0.795	↑
CD45(PTPRC)	0.017	1.728	0.789	↑
BDNF	0.022	1.691	0.758	↑
Osteocalcin (BGLAP)	0.034	1.685	0.753	↑
RYK	0.007	1.652	0.724	↑
PI16	0.045	1.643	0.716	↑
PDGFR-β(PDGFRB)	0.011	1.581	0.661	↑
SIGLEC9	0.026	1.553	0.635	↑
Lefty - A(LEFTY2)	0.048	1.548	0.630	↑
MDC(CCL22)	0.034	1.547	0.630	↑
INSL3	0.008	1.505	0.589	↑
C3a(C3)	0.039	0.648	-0.626	↓
FAK(PTK2)	0.034	0.618	-0.695	↓
HSP10(HSPE1)	0.019	0.609	-0.715	↓
Ubiquitin B(UBB)	0.044	0.609	-0.715	↓
BAI-1	0.030	0.515	-0.957	↓
MBL(MBL2)	0.003	0.508	-0.977	↓
TSLP R(CRLF2)	0.026	0.456	-1.132	↓
LIMPII(SCARB2)	0.012	0.315	-1.665	↓
IRF6	0.001	0.288	-1.796	↓

SAA1	<0.001	0.090	-3.480	↓

*a.k.a.: also known as.

AS: ankylosing spondylitis; ADA: adalimumab; FC: fold change.
