# Supplementary material for: Circulating Retinol-Binding Protein 4 as a Possible Biomarker of Treatment Response for Ankylosing Spondylitis: An Array-Based Comparative Study
Source: Front Pharmacol. 2020 Mar 10;11:231. doi: 10.3389/fphar.2020.00231 (PMC7076136; doi:10.3389/fphar.2020.00231)
Supplement: Supplementary file 6 [file Table_5.PDF]

Table S5. Subgroup analyses among healthy controls, AS patients with and without peripheral joint involvement

Protein name	pAS vs. HC		non-pAS vs. HC		pAS vs. non-pAS	
	P value	log2FC	P value	log2FC	P value	log2FC
SAA1	<0.001	4.286	0.004	2.518	0.028	1.768
ADAMTS-10	0.094	-0.954	0.029	-1.101	0.835	0.146
IRF6	<0.001	2.087	0.020	1.100	0.009	0.987
Osteocalcin	0.250	-0.624	0.011	-1.755	0.087	1.131
PDGFR-β	0.448	-0.363	<0.001	-1.613	0.056	1.249
RBP4	0.013	-2.923	<0.001	-3.464	0.079	0.541
ROR2	0.532	-0.311	0.009	-1.122	0.113	0.812

AS: ankylosing spondylitis; pAS, AS patients with peripheral joint involvement; non-pAS, AS patients without peripheral joint involvement; FC: fold change.
