# Supplementary material for: Circulating Retinol-Binding Protein 4 as a Possible Biomarker of Treatment Response for Ankylosing Spondylitis: An Array-Based Comparative Study
Source: Front Pharmacol. 2020 Mar 10;11:231. doi: 10.3389/fphar.2020.00231 (PMC7076136; doi:10.3389/fphar.2020.00231)
Supplement: Supplementary file 7 [file Table_6.PDF]

Table S6. Subgroup analyses among healthy controls, AS patients with and without hip joint involvement

Protein name	hAS vs. HC		non-hAS vs. HC		hAS vs. non-hAS	
	P value	log2FC	P value	log2FC	P value	log2FC
SAA1	<0.001	3.996	<0.001	3.152	0.776	0.844
ADAMTS-10	<0.001	-1.593	0.023	-0.794	0.239	-0.799
IRF6	0.002	1.700	<0.001	1.537	0.643	0.163
Osteocalcin	0.099	-1.159	0.037	-1.231	0.881	0.072
PDGFR-β	0.010	-1.281	0.062	-0.697	0.391	-0.584
RBP4	<0.001	-3.246	<0.001	-3.227	0.947	-0.019
ROR2	0.055	-0.924	0.104	-0.678	0.639	-0.246

AS: ankylosing spondylitis; hAS, AS patients with hip joint involvement; non-hAS, AS patients without hip joint involvement; FC: fold change.
